# Retrieval of ferromagnetic fragments from the lung using video-assisted thoracoscopic surgery and magnetic tool: a case report of combat patient injured in the war in Ukraine

**DOI:** 10.1186/s12245-023-00527-8

**Published:** 2023-08-24

**Authors:** Igor Lurin, Eduard Khoroshun, Volodymyr Negoduiko, Volodymyr Makarov, Serhii Shypilov, Volodymyr Boroday, Maksym Gorobeiko, Andrii Dinets

**Affiliations:** 1 National Academy of Medical Sciences of Ukraine, Kiev, Ukraine; 2 State Administrative Department, State Institution of Science “Research and Practical Center of Preventive and Clinical Medicine, Kiev, Ukraine; 3 Department of Thoraco-Abdominal Surgery, Military Medical Teaching Center of the Northern Region of Ministry of Defense of Ukraine, Kharkiv, Ukraine; 4 Department of Surgery #4, Kharkiv National Medical University, Kharkiv, Ukraine; 5 Department of Surgery, Institute of Biology and Medicine, Taras Shevchenko National University of Kyiv, Kiev, 03039 Ukraine

**Keywords:** War in Ukraine, Russo-Ukrainian war, Gunshot injury to lung, Gunshot injury to chest, Video-assisted thoracoscopic surgery, Surgical magnetic tool

## Abstract

**Background:**

Gunshot wounds injury to the thorax is common in armed conflicts or war, including the war of Russia against Ukraine. Injury to the chest is associated with a high mortality or physical disability due to damage to the lungs, heart, and major vessels.

The aim of this report is to demonstrate a case of successful management of severe gunshot injury to the lungs using video-assisted thoracoscopic surgery and magnetic tool for a combat patient injured in the war in Ukraine.

**Case presentation:**

A 51-year-old soldier of the Armed Forces of Ukraine received a gunshot injury due to shelling from artillery strikes in the Donbas battlefield area. After evacuation to Level II, a forward surgical team performed primary surgical debridement. Two hours after the injury, the patient was evacuated to the Level IV of medical care (Kharkiv). At Level IV, a CT scan showed penetrating gunshot wounds to the left part of the chest with injury to the upper lobe of the left lung with the presence of the 2 metal fragments of the artillery projectile with the size of 2.5 × 2.0 cm and 1.0 × 1.0 cm. These two fragments were removed by using video-assisted thoracoscopic surgery (VATS) using the inlet gunshot hole in the left lateral chest area, as well as the assistance of a magnetic tool.

**Conclusions:**

VATS and magnetic technologies should be considered for hemodynamically stable combat patients with a gunshot injury to the lungs in the ongoing war. Each combat patient could be treated by individualized approach such as using the wound canal as a scope port after primary surgical debridement of the wound and antibiotic prophylaxis.

**Supplementary Information:**

The online version contains supplementary material available at 10.1186/s12245-023-00527-8.

## Background

Gunshot injury to the chest is common in armed conflicts or warfare, including the war of Russia against Ukraine, which is ongoing since 2014 as hybrid warfare, and an active invasive phase has been started on February 24, 2022 [1, 2]. Injury to the chest is associated with a high mortality or physical disability due to damage to the lungs, heart, major vessels, etc. Since World War II, injury to the chest was and remained the common cause of up to 1/3 of deaths among combatants. Ongoing warfare in Ukraine is associated with the frequent use of multiple-launch rocket systems (MLRS) as well as various high-energy weapons causing severe injuries, limb amputations, bleeding, and lethal outcomes [3–5]. According to our clinical observations, gunshot injury to the lungs is the most frequent as compared to other locations, which is constituted up to 80% out of all other wounds to the chest (e.g., wounds to the pericardium, heart, major vessels) in the war in Ukraine since 2014. Gunshot injury to the lung is usually accompanied by damage to the ribs, severe bacterial contamination, and a high mortality rate (up to 37%) due to massive hemorrhage and acute respiratory failure [6]. The application of computed tomography (CT) scan is useful for the evaluation of both chest damage as well as a projectile trajectory in civil and combat conditions [7, 8]. Open thoracotomy is the method of choice in gunshot injuries to the chest, and video-assisted thoracoscopic surgery (VATS) is uncommonly applied to remove foreign bodies [8–13]. It is also important to mention that the Armed Forces of Ukraine and civil people are in permanent violation of international humanitarian law during the war by the Russian army because of such high-energy weapons as artillery strikes, cruise missiles, and drone attacks on medical facilities, causing significant problems for the safe evacuation of wounded to appropriate levels of medical care and interruption in supplying of medical goods [2, 14–17]. The management of all kinds of wounds is a difficult task for both civil people and military personnel under the abovementioned conditions. It is also worth mentioning that despite a sufficient experience in the management of gunshot injuries to the chest, it is sometimes a clinical challenge for choosing the most appropriate treatment approach for multiple gunshot injuries to the chest by high-energy weapons.

The aim of this report is to demonstrate a case of successful management of severe gunshot injury to the lungs using video-assisted thoracoscopic surgery and magnetic tool for a combat patient injured in the war in Ukraine.

## Case presentation

**Fig. 1:**
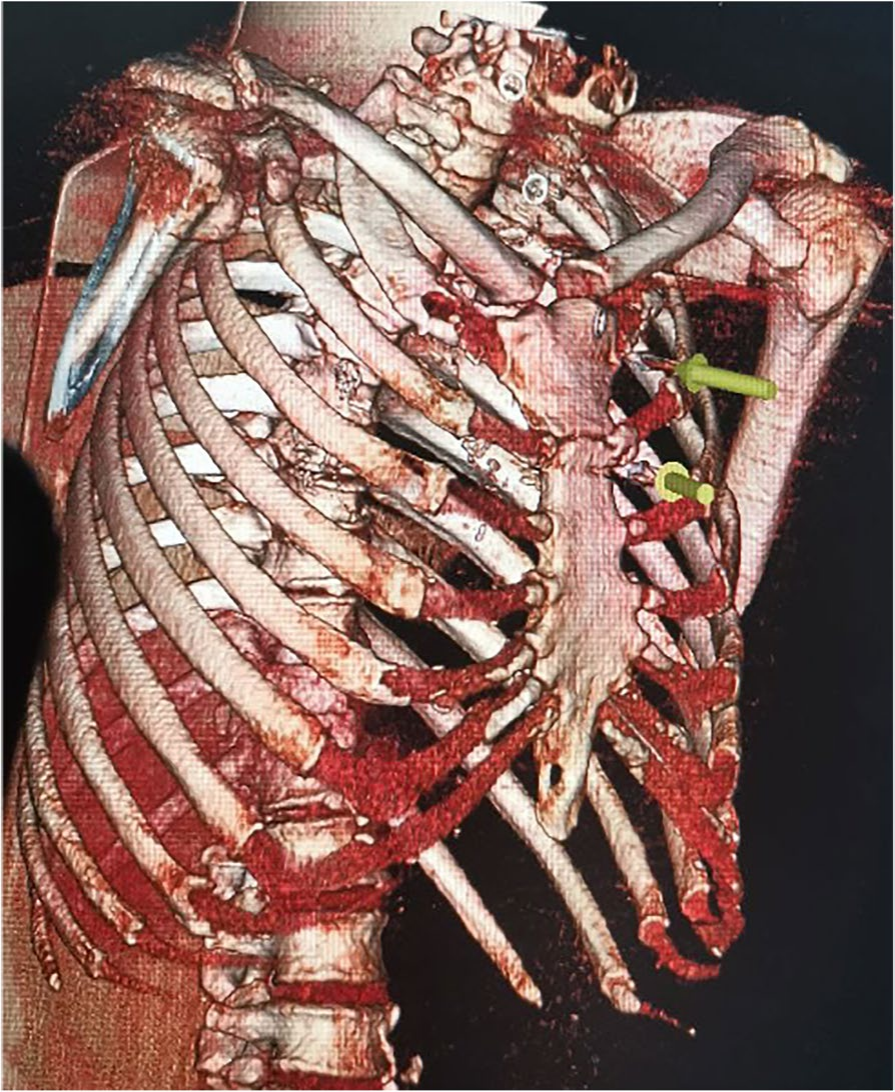
Illustration of 3D computed tomography scan of the chest showing localizations of two metal fragments in the lung (marked with two yellow pins)

**Fig. 2:**
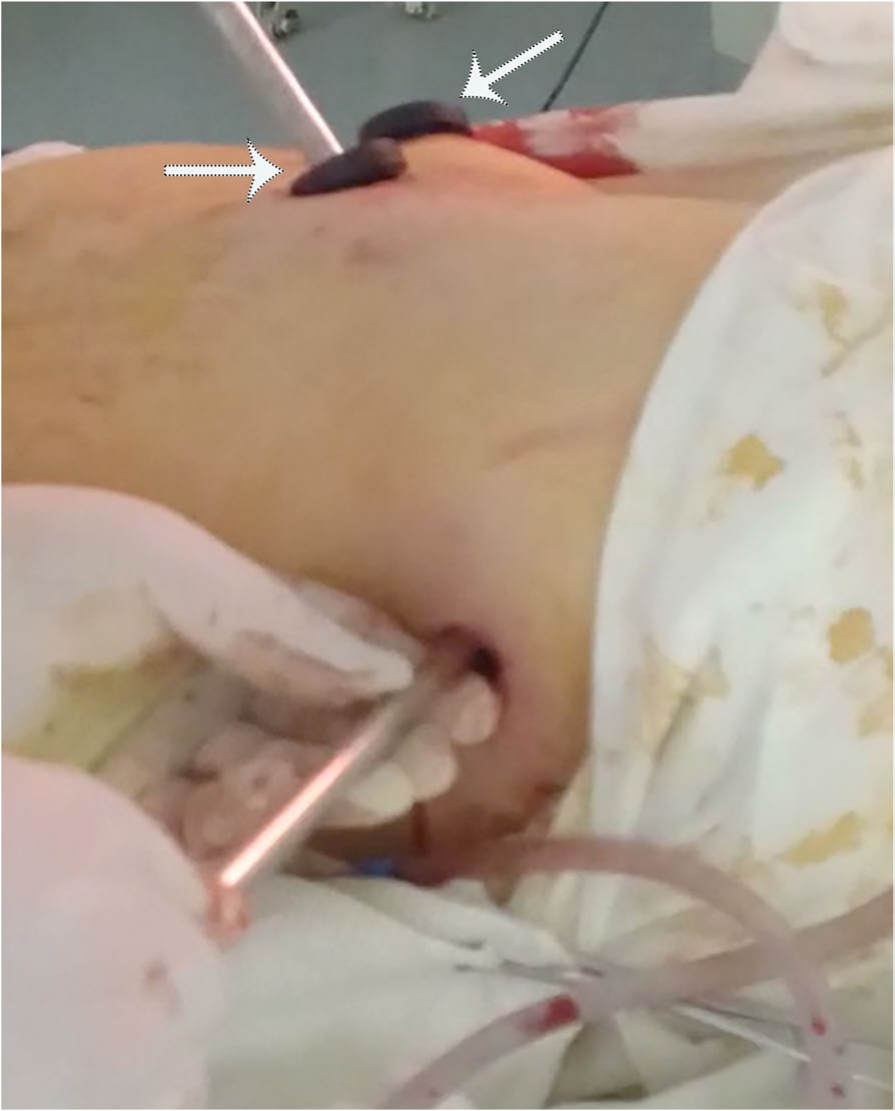
Intraoperative photograph showing inlet gunshot hole on the left side of the patient which is used as a thoracic port for video-thoracoscopic instruments. Two magnetic tools were used to fix the metal fragments in the lung before helping with its removal (marked with two arrows)

**Fig. 3:**
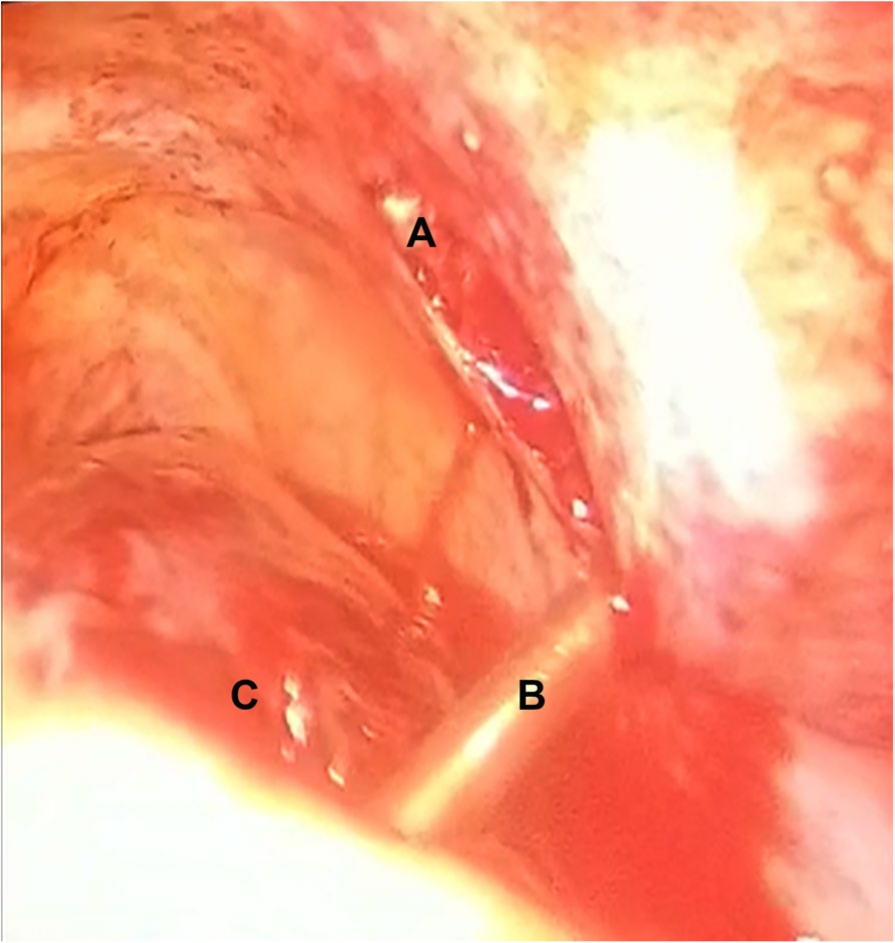
Intraoperative photograph of the thoracic cavity showing an internal view of the gunshot inlet hole (**A**), thoracoscopic instrument (**B**), lung (**C**)

**Fig. 4:**
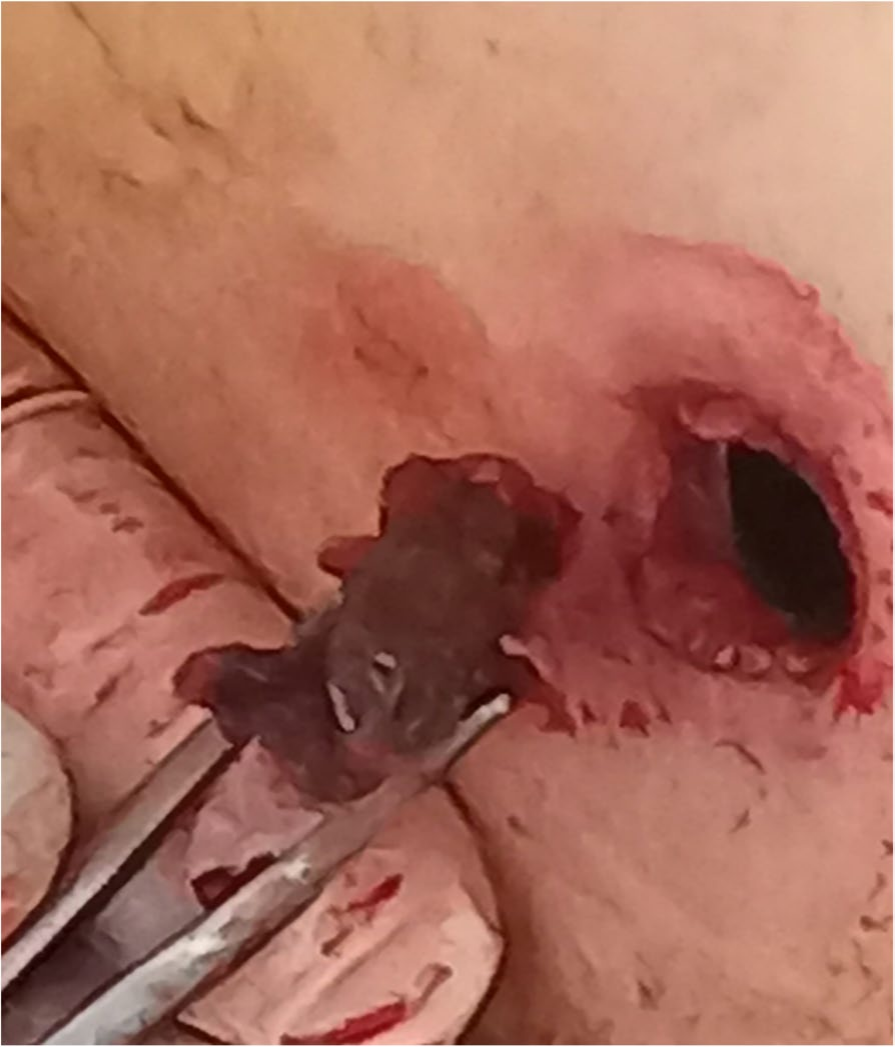
Intraoperative photograph of the removed metal fragment from the lung

A 51-year-old soldier of the Armed Forces of Ukraine received a gunshot injury due to shelling from artillery strikes in the Donbas battlefield area (East Ukraine). The shelling trauma was judged as severe because it was penetrating body armor and caused bleeding from the chest. The patient received medical aid at the place of injury according to the principles of damage control surgery and in line with the levels system of medical care as shown in our previous reports [2–4, 14, 16, 17]. One hour after the injury, the patient was evacuated to Level II, and forward surgical team (FST) performed primary surgical debridement of the gunshot wounds, including the application of drainage for chest decompression. Two hours after the injury, the patient’s condition was judged as severe, and the decision was made to evacuate him to the Level IV of medical care, which is the Military Medical Teaching Center of the Northern Region in Kharkiv. Upon admission to Level IV, the patient was subjected to a physical examination, presenting a wound of 3.0 × 2.0 cm in the left inguinal area along the middle inguinal line, and another wound of 1.5 × 1.0 cm was found in the area of the left shoulder blade. The weak breathing sounds were in the left parts of the chest. A spiral computed tomography (CT) scan of the chest was performed on a device with a tomograph step of 0.5 mm (Toshiba Activion 16, Japan). CT scan showed penetrating gunshot wounds to the left part of the chest with injury to the upper lobe of the left lung with the presence of the 2 metal fragments of the artillery projectile with the size of 2.5 × 2.0 cm and 1.0 × 1.0 cm. These two metal fragments showed radiodensity over 15,000 U by the Hounsfield scale, indicating the presence of ferromagnetic features (Fig. 1). Although the patient had severe wounds, he was in stable condition; therefore, the decision was made to perform video-assisted thoracoscopic surgery (VATS) for visual inspection of the pleural cavity as well as to remove metal fragments from the lung. An endoscopic magnetic tool was used to remove foreign bodies from the pleural or abdominal cavity, according to the previously described approach [18]. The video of the procedure is available as Additional file 1. The inlet hole in the left lateral chest area was considered convenient to use as a scope port for the magnetic tool (Fig. 2). An endoscopy magnetic retrieval tool was manually inserted into the chest cavity through the wound channel in the upper lobe of the left lung to the metal fragments under the video-endoscopic control. First, the smaller metal fragment (1.0 × 1.0 cm) was caught by the magnetic tool and removed using the same path from the wound channel. In the same manner, the larger metal fragment (2.5 × 2.0 cm) was also caught by the endoscopic magnetic tool; however, the size of that fragment did not fit the diameter of the wound channel, and the decision was made to temporarily keep the fragment at the level of the intercostal space by using magnetic control system (Fig. 3). The Mikulicz forceps were inserted into the area of the metal fragment (2.5 × 2.0 cm) location followed by fragment fixation and removed from the chest (Fig. 4). The patient was discharged from the hospital 2 weeks after the surgery and received 30 days of vacation according to the decision of the military-medical committee.

## Discussion

In this case report, we presented our clinical experience in the management of gunshot wounds with the application of VATS with magnet guidance to remove ferromagnetic fragments in combat patient. We believe this case report provides additional knowledge about the role of minimally invasive surgery and the magnets in the active war conditions accompanied by the violation of humanitarian law by the Russian army and in terms of limited medical resources in Ukraine [2, 14, 15, 17]. In regards to our previous reports, management of gunshot injury to the lungs is presented for the first time in the patient injured during the active (invasion) period of the Russo-Ukrainian war. This study is also supported by previous reports of the possibility to apply modern technologies and tools in conditions of limited medical recourses and high risk of strikes by high-energy weapons [2, 14, 15, 17]. Limited medical resources were and remained a common problem for healthcare in Ukraine due to various causes, including bad planning [19]. However, military and civil surgeons can diagnose and consider the management of such a severe vascular injury even in unstable combat conditions and available resources.

Similar to other reports, in this study, we have shown the utility of using a chest CT scan for evaluation of the damage to soft tissues, bones, mediastinum, pleura, and lungs [7, 8]. The accuracy and sensitivity of the CT scan are high to detect foreign bodies of any localization, signs of hemothorax or pneumothorax, and intrapulmonary hematoma. CT scan showed its utility in war conditions and specifically for gunshot injury to determine the trajectory of the wound channel and to characterize in detail the changes in the anatomical structures of the chest cavity organs due to injury. By using a CT scan a short time after the injury, it is possible to obtain topographical and anatomical characteristics of the wound channel, detect foreign bodies, determine their relationships with surrounding organs and structures, and obtain comprehensive data on the nature of damage to the chest organs and adjacent cavities. The abovementioned approach is associated with choosing of most rational treatment tactics, optimal access for surgical intervention and drainage area, and the choice to apply minimally invasive treatment methods such as VATS and magnets. The limitation exists the wide application of CT scan in Ukraine within the area of 20–40 km from the front line due to the high risk of MLRS or autonomous pusher-prop drones’ application, and risk is lesser but still exists due to the risk of cruise missile attacks in the hospitals at Levels III and IV.

It is well known that the presence of the large foreign fragments in the lungs is an indication for surgical treatment [8, 9]. We used a gunshot wound channel as a path for the retrieval of the metal fragments, which is in contrast to other reports. Usually, the wound channel is not used as a thoracic path in case of penetrating wounds to the chest due to the possible presence of microbial contamination. However, in this particular case, we decided that the wound channel would play the most optimal role as a thoracic port because it was the shortest path to remove the foreign body, and the patient was under antibiotics therapy. The combination of VATS with the guidance of a magnetic tool made it possible to remove metal fragments minimally invasive and without conversion to the thoracotomy.

Similarly, Kakamad et al. and Khalil et al. also showed the utility of VATS to remove bullets from the chest [9, 13]. However, in contrast to that studies, we decided to use also magnet, considering the ferrous nature of the gunshot fragments and the availability of special magnetic tools.

In line with Essa et al., we also decided to use VATS in war-related shrapnel injury to the lungs, considering hemodynamically stable patients and faster recovery after the surgery [20]. As also suggested in other reports, we also decided to use a surgical instrument (Mikulicz forceps) to remove the large metal fragment, indicating such an approach to be a useful part of the VATS application [21].

It is also worth to mention that VATS is infrequently performed for the foreign bodies in the lungs and bronchi. The majority of reports of VATS or conventional thoracotomy are related to the removal of foreign bodies in pediatric patients, whereas adult patients with gunshot trauma are treated by conventional thoracotomy [22].

## Conclusions

VATS and magnetic technologies should be considered for hemodynamically stable combat patients with a gunshot injury to the lungs in the ongoing war. Each combat patient could be treated by individualized approach such as using the wound canal as a scope port after primary surgical debridement of the wound and antibiotic prophylaxis.

### Supplementary Information

**Additional file 1.** Intraoperative video of using video-assisted thoracoscopic surgery (VATS) and surgical magnetic tool to remove metal fragments.

## Data Availability

All data regarding this case report has been reported in the manuscript. Please contact the corresponding author in case of requiring any further information.
